# An application of programmatic assessment for learning (PAL) system for general practice training

**DOI:** 10.3205/zma001133

**Published:** 2017-11-15

**Authors:** Lambert Schuwirth, Nyoli Valentine, Paul Dilena

**Affiliations:** 1 Flinders Universität, Adelaide, Australia; 2 Universität Maastricht, Mastricht, The Netherlands; 3 Chang Gung Universität, Taiwan; 4 Uniformed Services University, USA; 5 Sturt Fleurieu GP, GPEx, Australia

**Keywords:** Assessment, Programmatic assessment, General practice training

## Abstract

**Aim: **Programmatic assessment for learning (PAL) is becoming more and more popular as a concept but its implementation is not without problems. In this paper we describe the design principles behind a PAL program in a general practice training context.

**Design principles: **The PAL program was designed to optimise the meaningfulness of assessment information for the registrar and to make him/her use that information to self regulate their learning. The main principles in the program were cognitivist and transformative. The main cognitive principles we used were fostering the understanding of deep structures and stimulating transfer by making registrars constantly connect practice experiences with background knowledge. Ericsson’s deliberate practice approach was built in with regard to the provision of feedback combined with Pintrich’s model of self regulation. Mezirow’s transformative learning and insights from social network theory on collaborative learning were used to support the registrars in their development to become GP professionals. Finally the principal of test enhanced learning was optimised.

**Epilogue: **We have provided this example explain the design decisions behind our program, but not want to present our program as the solution to any given situation.

## Zusammenfassung

**Ziel: **Die programmatische Leistungsmessung bei Lernvorgängen (programmatic assessment for learning=PAL) wird als Konzept immer beliebter, jedoch ist dessen Umsetzung nicht unproblematisch. In dieser Arbeit beschreiben wir die Design Prinzipien, die einem PAL-Programm im Kontext der Facharzt-Ausbildung von Allgemeinmedizinern zugrunde liegen.

**Design Prinzipien: **Das PAL-Programm wurde entworfen, um die Aussagekraft von Informationen zur Beurteilung der Leistungen eines Weiterzubildenden zu optimieren und diese dazu zu bewegen, diese Informationen zu nutzen, das eigene Lernen selbst zu steuern. Die Hauptprinzipien des Programms sind kognitivistischer und transformativer Art. Die wesentlichen kognitiven Prinzipien, die wir dabei verwendeten, förderten das Verständnis tieferer Strukturen und stimulierten den (Wissens-)Transfer, indem die Weiterzubildenden kontinuierlich dazu angehalten wurden Praxiserfahrungen mit Hintergrundwissen zu verbinden. Ericssons Prinzip der bewussten Praxis wurde im Hinblick auf das Feedback zu Leistungserhebungen integriert und mit Pintrichs Modell der Selbststeuerung kombiniert. Mezirows transformatives Lernen und die Einblicke in die Sozialnetzwerktheorie zu kollaborativem Lernen wurden dazu verwendet, um die Weiterzubildenden in ihrer Entwicklung hin zu professionellen Fachärzten der Allgemeinmedizin zu unterstützen. Zu guter Letzt wurde noch das Prinzip des Test-basierten Lernens verbessert.

**Epilog: **Wir präsentieren dieses Beispiel, um die Design Entscheidungen, die unserem Programm zugrunde lagen, zu erläutern; jedoch möchten wir unser Programm nicht als (Patent-)Lösung für jede beliebige Situation verstanden wissen.

## Background

Programmatic assessment for learning (PAL) is rapidly gaining popularity around the world [1], [2], [3]. This is quite surprising, because the concept is fundamentally different to what has been the custom in assessment in the past. Traditionally, assessment focused almost entirely on determining whether a student had learnt enough to prevent not-yet-competent students from progressing. Where there was an influence of assessment on student learning, it was mainly used from a behaviourist viewpoint; that is, passing the assessment was the proverbial “carrot” and failing was the “stick”. However, the way assessment influences student learning is far more complex than this. Cilliers et al. for example showed the myriads of interactions between sources of impact on learning, the mechanisms by which learning is impacted and the possible consequences [4], [5]. This understanding of the relationship between assessment and student learning behaviour is important in using assessment specifically to direct student learning in a more meaningful way. This is the main purpose of assessment ***for*** learning [6], [7].

In order for assessment to drive student learning in a more meaningful way, the student has to be enabled to obtain meaningful information about their own performance and use this for their future learning. This is where the programmatic aspect of PAL comes in [8]. Just providing a score and a pass-fail decision, for example, does not inform the student sufficiently about their strengths and weaknesses. So, it does not help them in formulating more specific and concrete learning plans. Also in many traditional assessment programs, combining information is based on the format of the assessment; in an OSCE, for example, performance on an abdominal examination station is combined with performance on a knee examination. In PAL, information is combined across different assessment methods to make it more meaningful. This principle is perhaps best illustrated using a clinical example [9]. When we combine our patient’s complains about fatigue, thirst and frequent urination with physical examination findings such as poorly healing wounds and absent peripheral arterial pulsations, and with the numerical value of 32 mmol per litre for the blood glucose it easily adds up to “diabetes mellitus”. We would not consider telling a patient that their glucose level is far too high but fortunately their potassium level is far too low so on average they are okay, which would be the clinical equivalent of combining the performance on an abdominal examination patient with the performance on a knee examination station. In programmatic assessment we therefore aim to combine information across assessment methods in the same way to diagnose “dyscompetence”. Of course the disease “dyscompetence” does not exist but we use the term merely to illustrate principle.

An additional feature of programmatic assessment is the concept of proportionality. This means that the stakes of the decisions made about a student or learner have to be proportional to the credibility of the underlying information. So, single observations or single assessments can be used for feedback but not for high-stakes decisions. In programmatic assessment, all single observations or assessments are collected and collated over time until sufficient information is available to make a high-stakes decision [10]. This again, is quite similar to daily clinical practice; we are comfortable with making a simple diagnosis – for example an upper respiratory tract infection – on the basis of little information but for a high-stakes diagnosis – such as a malignancy – we want to rely on multiple sources of diagnostic information (lab values, imaging, pathology, et cetera).

So logically, programmatic assessment is a longitudinal approach to assessment in which the outcomes of many formal and informal assessments are collected on a continual basis, for example in a portfolio. Typically, the learner and a staff member – often called a mentor or coach – meet at regular intervals to discuss the learner’s progress and their concrete learning goals. And at the end of the study phase, all information is used to decide whether the learner is allowed to progress or not. Importantly, the learner makes an analysis based on all the information available to him or her, and formulates concrete learning goals before meeting with their mentor or coach. As such, the coach is able to give a prognosis during the phase of the most likely outcome. The concept of programmatic assessment has been described in various publications [1], [7], [8], [10].

Typically, when the concept of PAL is explained – especially to healthcare providers – it feels intuitively right and people are willing to accept the concept, but implementation is not at all easy. There are several reasons for this. The first and probably most important reason, is the fact that it is a fundamental change in thinking about the role of assessment. Fundamental changes to any discipline just need time to find their way from theory to practice. A conceptually different way of thinking about education, such as problem-based learning, has taken many years and even decades before it has become widely accepted. A second reason concerns the logistical changes needed for the implementation. In PAL the whole assessment program is explicit and therefore the associated investment in time and costs are overt, whereas in many traditional assessment programs costs are generally more covert. That makes a cost comparison quite difficult and easily result in a negative perception towards PAL. The third reason lies most likely in what Vosniadou calls naïve frameworks or naïve theories [11]. Through our experiences in the world we develop our own views on how the world works and it is very difficult to change these beliefs. They can complemented by more formal theories, but they never really disappear. This also happens with education. Our views on what education is and how it should be organised have been shaped by our lengthy experience as learners ourselves, and although they can be complemented by formal training – such as staff development or teacher training –they never completely disappear. So when an attempt is made to implement PAL the naïve beliefs still continue to influence the various detailed design decisions.

Some of the more intuitive approaches to deal with beliefs and the management of such a change concern careful identification of stakeholders and their roles and careful communication with them. This communication needs to be open and continuous but also agile in that it relates to different stakeholders with different arguments and explanations; ranging from evidence from research to rhetorical conviction. In addition, one of the factors that can help in this process is a description of implementations somewhere else; as a demonstration that the concept can be translated to an actual practice. The purpose of this paper is to provide such a proof of concept. In the Consensus statement and recommendations from the 2010 Ottawa conference [12] the ideographic description or educational case report is seen as an important type of research as long as it connects the described practice with the underlying theoretical concepts so that it allows the readers to understand the design decisions and adapt them to their own context. In medical education, this ‘adaptability’ is considered more helpful than mere replicability of findings [13]. This paper therefore presents an educational case study.

## Context

GP365 is a general practice training program in South Australia and Western Australia, which has been developed by Sturt Fleurieu Education and Training in collaboration with the Flinders University Prideaux Centre for Research in Health Professions Education. It is a one-year curriculum in the context of a three or four-year training program and it is run for all GP registrars in South Australia and Western Australia. During this year, GP registrars (residents) follow the GP365 program which supports them through linking their practice experiences to background knowledge, skills and understanding. During this year, GP365 provides registrars with background reading material, assignments, a supervisor, a medical educator and a peer group to work with. On a continual basis registrars receive feedback from their supervisors, from medical educators and from their peers. In addition, they are given formative tests on relevant knowledge and application of knowledge. During their training registrars build a portfolio which will eventually contain evidence of all feedback from: directly observed patient consultations, reviewed videoed consultations, critical case analysis write-ups, a clinical audit they have performed, professionalism, activities in their peer group, multi-source feedback, mid and end term assessments, the results of their formative tests with their own analyses and written feedback from their medical educator. 

In total, this program may not seem innovative or different to what is done in many postgraduate training contexts, but there are differences which we will explain below.

## Design principles

The most important design principle behind GP365 is the problem of transfer and understanding of the so-called “deep structure” [14]. Registrars, during their training, see a huge variety of individual patient cases, but to become an expert it is important to understand similarities and differences between these cases; thus to build transfer [15]. The literature describes the importance of decontextualizing, understanding first principles and recontextualising (applying those principles in another case) for the development of transfer and expertise [15], [16]. Therefore, in GP365 the assessment seeks to support the registrar in meaning making of these individual experiences, for example by requiring them to relate basic medical sciences and background clinical knowledge to their individual patient experiences. This is typically what the critical case analysis write-ups focus on. The registrar chooses a patient for their critical case analysis write up, but has to be able to explain why they see that particular patient as most relevant to their own learning. The registrar also defines their own concrete learning goals and then studies the necessary background information to obtain a complete understanding of the clinical case and its management. As “evidence” of this learning the registrar produces three case based multiple-choice questions backed up by a literature reference. He or she then submits the case write up and questions. The medical educator reads the clinical case write up and provides ample feedback which the registrar has to implement in a revised version of the case write up. As such this is an activity that requires the registrar to make optimal meaning of what they have experienced during their practice but with evidence of the related learning. This evidence is reviewed and will be a mandatory part of their portfolio – as are all sorts of evidence – and contribute to the final decision.

The second design principle is the aggregation of information across different assessment parts. An example of such connection starts with the critical case analysis write-ups. As described above, the registrar receives feedback on all their write-ups which they have to implement and revise. This is an application of Ericsson’s principle of deliberate practice [17], [18]. The multiple-choice questions that each registrar generates are collected into an item bank. From this bank, periodically, progress tests are constructed and presented to the registrars, which they can sit during a predefined time window using the electronic learning system. The test items are then released and the registrars are required to critique at least three questions; preferably those questions they find most contentious. When they critique questions, they have to provide copies of the relevant scientific literature supporting their critique. The idea behind this is to optimise the influence of test enhanced learning by asking the registrars to critically review the items and their own responses [19] and has been used in progress testing in various settings [20], [21]. These critiques are discussed in a peer group meeting. After this exchange of critiques between the members of the group, the group is expected to produce a consensus of the questions that they find most contentious and with a summary of their critique. Only then will the registrars receive their scores on the test, which they can then analyse and use in their portfolio. The design principle behind the group meetings is to foster the development of informal peer networks. Registrars may be practising in remote areas, and many have limited or no colleagues their own age or experience in their practice which they feel they can relate too. The literature shows that the possession of informal networks is important for receiving information and learning [22], [23]. A further included principle from transformative learning theory relates to making registrars aware that not all which is written is necessarily unambiguously true, and that tolerance for uncertainty is part of practice [24]. This is an illustration of how the assessment programme leads the registrars to integrate information from the assessment in a more meaningful way across instruments, so as to optimise the ‘constructivist’ drivers of the assessment on learning.

A third principle is an increase in self responsibility for learning. Generally, after graduation learners are expected to be able to take control of their own learning and assessment. Unfortunately, this is not always the case. One of the problems with CME, for example, is that people tend to follow courses in those areas there are already good at [25]. Assessment for learning should actually equip learners with the ability to analyse their strengths and weaknesses, to translate these into specific learning goals and to actually make that learning occur. For most people, self-regulated learning does not come naturally and it requires development and guidance. Paul Pintrich’s model is helpful as it distinguishes activities such as: “forethought, planning and activation”, “monitoring”, “control” and “reaction and reflection”; each of which requires learners to manage their cognition, motivation, behaviour and context [26]. Of course, having regular meetings and requiring the registrars to constantly analyse their own progress, strengths and weaknesses, and by asking them to formulate concrete do-able learning goals, the elements of “forethought, planning and activation”, “monitoring”, “control”, and “reaction and reflection” are built into the assessment system. Registrars who fail to undertake or do not complete these self-regulation activities in a comprehensive manner, are required to re-do the activities and will eventually not be allowed to progress. By providing the registrars with feedback, a peer group, a supervisor and a dedicated medical educator, the program supports the registrars’ motivation and behaviour, and by providing them with ample information through the assessment it supports their cognition. Their relationships with their supervisor, medical educator and their peer groups provide support around learning how to navigate their current and future complex context. Registrars who do not progress as desired will have to follow remediation. But, they themselves will have to take action and design their own remediation, of course with the support and permission of their supervisor and medical educator. As such, they will have to take full responsibility for their own learning, like they will have to do after graduation.

A fourth principle is the longitudinality of the programme. All the information about the registrar’s performance is collected in a portfolio which is discussed periodically with their supervisor and/or medical educator. Initially, when the information in the portfolio is still ‘thin’ mostly more formative feedback is given, when the information becomes richer serious suggestions for intervention and remedial activities are given and the final decision whether or not the registrar is ready to progress to the next phase is always based on the full and rich information. 

A final design principle is adaptation of the program to the local context. It is unlikely that any approach in medical education which works well in one country or context could be simply applied in another country. In order for education to be successful it has to link with expectations and cultural determine of its environment. In GP365 it was therefore important to realise that Australia is a vast country with many remote areas. GPs play an important role in the Australian healthcare context and almost always they are the first port of call; moreover, they are often the only port of call. This puts a specific stress on educational programs to educate GPs who are optimally equipped to work individually and safely. Therefore, elements such as self-directed learning, accountability, dealing with uncertainty and tolerance for ambiguity features so prominently in the program, for example like the peer group meetings – the so-called mini releases –, the ongoing feedback on professionalism and the frequent meetings with experienced supervisors and medical educators. This is woven into the program in all kinds of aspects to ensure that the program meets the Colleges’ requirements of “safe independent practitioner” and “college exam readiness”.

## Epilogue

The program has now been running for two years and it is too soon to provide extensive information as to its success with respect to the quality of graduates. Early results do suggest that the pass rates on fellowship exams are far above the national average but before a clear causal relationship with the PAL approach can be argued for more data are needed. That was not the intent of this paper and although there are anecdotal indications that programmatic assessment for learning is effective, the whole approach is still too young to have produced a sufficiently credible body of evidence. So the jury is still out. Moreover, we feel that any success in our context would not mean that the exact same program would have the same success in another context. That’s why we have focused on providing examples on how the concept of programmatic assessment for learning has influenced design decisions around GP365 assessment program. We hope that our explanation of the underlying principles and the description of how they have influenced our design decisions are helpful for anyone who is considering changing or rebuilding programmatic assessment for learning program. 

## Competing interests

The authors declare that they have no competing interests. 
